# Romanian Journal of Ophthalmology is indexed in PMC and PUBMED. More to come on the indexing processes soon

**Published:** 2018

**Authors:** Consuela-Mădălina Gheorghe

**Affiliations:** * Philologist, Authorized translator

Three years have passed since *Romanian Journal of Ophthalmology* has changed its name from Oftalmologia and begun being published in English. At that time, the aim was to obtain a better visibility and quotation, which has been partly achieved. 

Starting 2017, a contract has been signed with The National Library of Medicine (NLM), thus, *all the articles* up to date *have been indexed in PMC and PubMed*. *PubMed Central* (PMC) is a free digital repository that archives publicly accessible full-text scholarly articles that have been published within the biomedical and life sciences journal literature (source: https:// en.wikipedia.org/ wiki/ PubMed_Central). *PubMed* is a free search engine accessing primarily the MEDLINE database of references and abstracts on life sciences and biomedical topics (source: https:// en.wikipedia.org/ wiki/ PubMed). In other words, we managed to fulfill our desideratum of making our journal popular and *increase its visibility*. Of course, this change has brought more value to our journal, now being indexed in an international database. In addition, we welcome scientists, academic personalities, etc., from Romania and the whole world to send their articles to be published in *Romanian Journal of Ophthalmology*. 

The process of indexing was a lengthy one, starting with the *indexing of the abstracts of the articles in MEDLINE*, continuing with the conversion of all the articles in a specific format (XML) and their uploading on PMC platform and in PubMed database. 

Regarding process of indexing, *MEDLINE* indexers describe the content of biomedical articles by assigning subject terms to them. These subject terms are selected from the controlled vocabulary, Medical Subject Headings (MeSH). The *MeSH* terms assigned to an article appear on the bibliographic citation in *PUBMED*. They can be used in PUBMED searches and retrieval (source: https:// www.nlm.nih.gov/ bsd/ indexing/ training/ INT_010.html).

The *MeSH* controlled vocabulary, developed at *NLM (National Library of Medicine*), contains over 27,000 terms covering life sciences, medicine, as well as many other areas. MeSH vocabulary reflects the progress in biomedical sciences. Every year, several hundred new terms are added to the MeSH vocabulary, and some existing terms are modified. In addition to the MeSH descriptors, MEDLINE indexers also use 79 topical subheadings or qualifiers (source: https:// www.nlm.nih.gov/ bsd/ indexing/ training/ INT_010.html).

For the full participation in PMC, which is a full text archive, a journal must meet the scientific quality standards established by the National Institutes of Health (NIH) and National Library of Medicine. The members of the latter will decide if the scientific and editorial scope and quality of a journal merit its inclusion in PMC. Moreover, the opinions of expert consultants will also count in the final evaluation of the journal (source: https:// onlinelibrary.wiley.com/ doi/ pdf/ 10.1002/ rth2.12047).

The journal must also meet the technical standards of the database by providing high-quality digital files (full text articles in XML format) (source: https:// onlinelibrary.wiley.com/ doi/ pdf/ 10.1002/ rth2.12047). 

The PMC application requires that a journal publish at least 25 peer-reviewed research articles before being indexed (source: https:// onlinelibrary.wiley.com/ doi/ pdf/ 10.1002/ rth2.12047). 

Regarding the inclusion of a journal in MEDLINE, there is a condition that a journal has been publishing for 12 months and has published at least 40 articles. The more content the journal has published, the stronger the application. The journal needs to appear sustainable by publishing quality content at a regular rate. Other elements taken into consideration include scope and coverage of the journal, quality of content and editorial work, production quality, and audience (source: https:// onlinelibrary.wiley.com/ doi/ pdf/ 10.1002/ rth2.12047).

MEDLINE requires that all published articles include declarations of conflict of interest by authors, confirmation that informed consent was sought from research subjects, and that animal welfare was taken into consideration (source: https:// onlinelibrary.wiley.com/ doi/ pdf/ 10.1002/ rth2.12047). 

What sets MEDLINE apart from the rest of PubMed is the added value of using the NLM controlled vocabulary, Medical Subject Headings (MeSH®), to index citations. PubMed has been available since 1996. Its more than 27 million references include the MEDLINE database plus different types of citations. PubMed citations often include links to the full-text article on the publishers’ Web sites and/ or in PMC and the *Bookshelf*. MEDLINE is the largest subset of PubMed (source: https://www.nlm.nih.gov/pubs/factsheets/dif_med_pub.html).

This indexing has been greatly appreciated so far by the academic community and we hope that this will result into more articles of Romanian and international authors being published in the future, taking into account that *we intend to index the journal in many other international databases, including Thomson Reuters (ISI)*. 

However, in order to succeed, we need the help and collaboration of all the authors who send their articles to be published in the journal. In addition, *before submitting, an author should be well acquainted with the editorial recommendations for writing an article* (information that can be found in the Guidelines for Authors section both in the printed version and on the journal’s website – *www.rjo.ro*). In addition, when writing an article the authors should properly mention the complete name and surname, address of the corresponding author, etc., and they should also comply with the IMRAD form: introduction, methods, results, discussion and conclusion, including acknowledgements, disclosures and all the *references, which should be all cited with no exception and at all times*. 

It was a tremendous effort for us to get where we are, but we must continue, improve, be better, and the rewards will surely be the ones expected. We have managed to publish an issue at every three months, which means that on one hand, we have kept our promise and on the other, the ophthalmology field is still interesting for many authors. We are very confident that together with the Romanian Society of Ophthalmology and the ophthalmological academic community, Romanian Journal of Ophthalmology can be the best journal in this field in Romania and abroad.

The steps taken so far have required and are still requiring the further improving of the quality of the published articles. 

Our recommendation to the authors, readers, members of the academic community, etc., is to get more involved in the effort of promoting the journal both in Romania and in the whole world and to encourage their peers to write and send articles to be published in our journal, to achieve visibility.

**Assist. Prof. Gheorghe Consuela-Mădălina, PhD,** **Philologist, Authorized translator**

